# Shedding of Klotho: Functional Implications in Chronic Kidney Disease and Associated Vascular Disease

**DOI:** 10.3389/fcvm.2020.617842

**Published:** 2021-01-28

**Authors:** Valeria Saar-Kovrov, Marjo M. P. C. Donners, Emiel P. C. van der Vorst

**Affiliations:** ^1^Department of Pathology, Cardiovascular Research Institute Maastricht, Maastricht University Medical Centre, Maastricht, Netherlands; ^2^Institute for Molecular Cardiovascular Research, Rheinisch-Westfälische Technische Hochschule (RWTH) Aachen University, Aachen, Germany; ^3^Interdisciplinary Centre for Clinical Research, Rheinisch-Westfälische Technische Hochschule (RWTH) Aachen University, Aachen, Germany; ^4^Institute for Cardiovascular Prevention, Ludwig-Maximilians-University Munich, Munich, Germany; ^5^German Centre for Cardiovascular Research, Partner Site Munich Heart Alliance, Munich, Germany

**Keywords:** Klotho, a disintegrin and metalloprotease, ectodomain shedding, chronic kidney disease, vascular disease

## Abstract

α-Klotho (Klotho) exists in two different forms, a membrane-bound and soluble form, which are highly expressed in the kidney. Both forms play an important role in various physiological and pathophysiological processes. Recently, it has been identified that soluble Klotho arises exclusively from shedding or proteolytic cleavage. In this review, we will highlight the mechanisms underlying the shedding of Klotho and the functional effects of soluble Klotho, especially in CKD and the associated cardiovascular complications. Klotho can be cleaved by a process called shedding, releasing the ectodomain of the transmembrane protein. A disintegrin and metalloproteases ADAM10 and ADAM17 have been demonstrated to be mainly responsible for this shedding, resulting in either full-length fragments or sub-fragments called KL1 and KL2. Reduced levels of soluble Klotho have been associated with kidney disease, especially chronic kidney disease (CKD). In line with a protective effect of soluble Klotho in vascular function and calcification, CKD and the reduced levels of soluble Klotho herein are associated with cardiovascular complications. Interestingly, although it has been demonstrated that soluble Klotho has a multitude of effects its direct impact on vascular cells and the exact underlying mechanisms remain largely unknown and should therefore be a major focus of further research. Moreover, functional implications of the cleavage process resulting in KL1 and KL2 fragments remain to be elucidated.

## Introduction

α-Klotho (Klotho) is a type I transmembrane protein which is highly conserved among human, mouse, and rat (up to 94% homological sequence) (1) and primarily expressed in the kidney, in both proximal and distal tubuli, though some expression has been shown in choroid plexus, parathyroid gland and sinoatrial node (2). Membrane-bound Klotho plays an important role in a wide range of physiological and pathophysiological processes [as recently reviewed in (3)]. For example, Klotho has not only been demonstrated to play an important role in renal function (4) and controls the brain-immune system interface (5). The membrane-bound Klotho protein forms a complex with the fibroblast growth factor receptors (FGFR), which is crucial for the binding of FGF23 (6). FGF23 exerts several endocrine functions, like regulating phosphate, calcium, and vitamin D homeostasis (7). Dysregulation of this FGF23-Klotho axis is not only associated with chronic kidney disease (8), but also with vascular and skeletal anomalies which are mainly caused by an altered phosphate turnover [as reviewed in (9)]. This abnormal phosphate regulation is the mechanism by which deficiency of FGF23 and Klotho is associated with accelerated aging (6, 10), which can be rescued by low phosphate diet feeding to restore the phosphate balance (11).

Besides this membrane-bound form, Klotho is also released in soluble form. This soluble Klotho is detectable in cerebrospinal fluid, after being shed from the choroid plexus, and in urine and blood, after being mainly shed from the kidney (12, 13). Soluble Klotho has not only a local impact on renal function but also systemic effects on the cardiovascular system (see chapter 3). Initially this soluble form was believed to arise from both proteolytic cleavage, a process called shedding, as well as secretion of an alternatively spliced Klotho variant. This spliced variant has a 50 bp insertion containing an in-frame stop codon, resulting in a truncated Klotho protein (14). Interestingly, this spliced, truncated version of Klotho has not been detected *in vivo* thus far (3, 13, 15). Recently, Mencke et al. described that this alternatively spliced variant is subjected to nonsense-mediated mRNA decay and therefore not secreted (15). Soluble Klotho thus solely arises from the shedding process, which will be further elaborated on in the next section.

## Klotho Shedding

Various type I transmembrane protein, like Klotho, can be cleaved by a process called shedding, which releases the ectodomain of the transmembrane protein (16). In this manner, shedding is a post-translational modification that controls the levels and function of hundreds of membrane proteins. Alpha secretases [e.g., “a disintegrin and metalloprotease” (ADAM)] as well as beta-secretases [e.g., “beta-site APP cleaving enzyme” (BACE)] have been described as the main sheddases, although in recent years a broader range of proteases has been identified to play a role in protein shedding (16).

**Figure 1:**
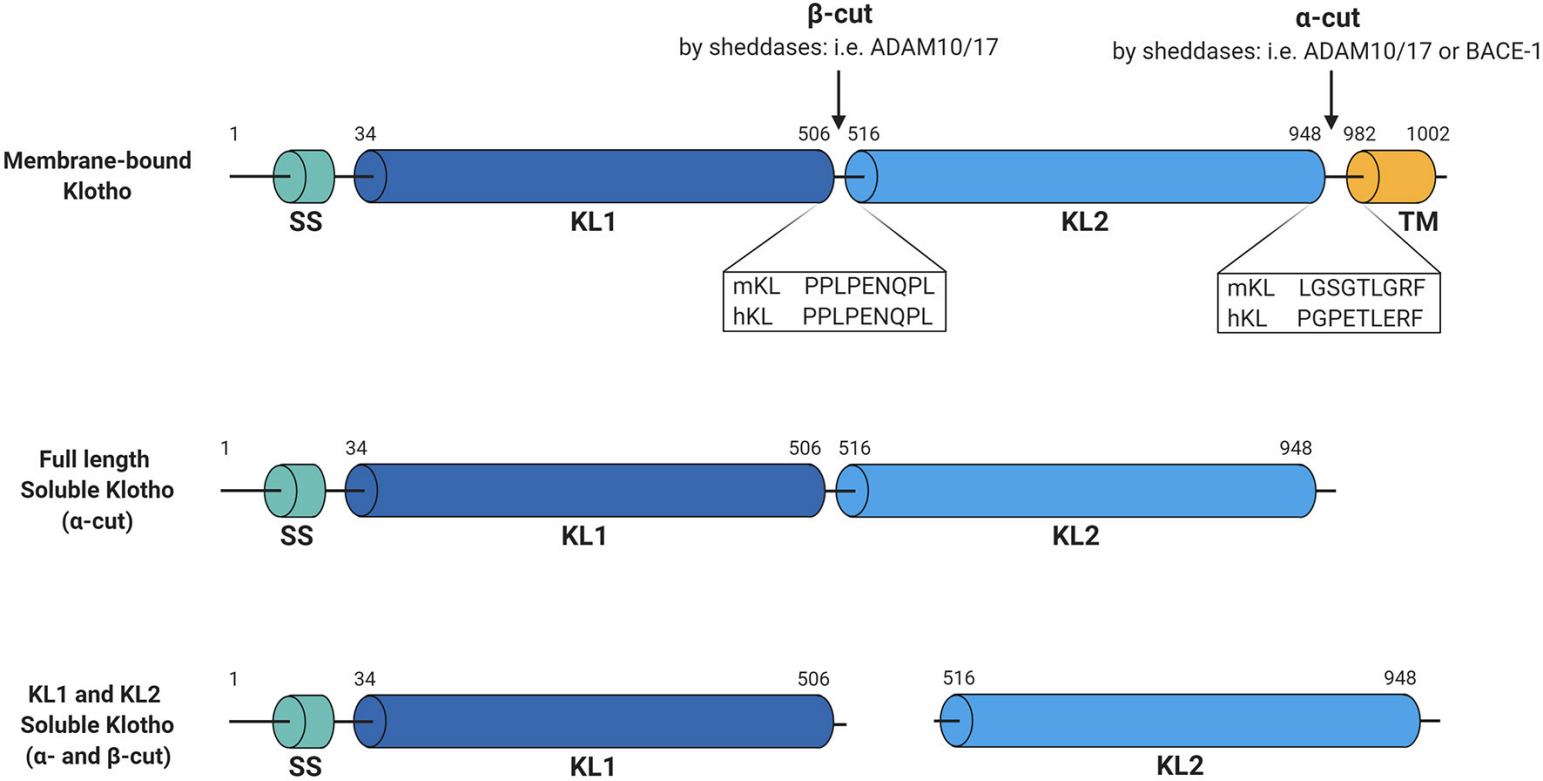
Schematic overview of Klotho structure and cleavage. Membrane-bound Klotho consists of four main domains, being the signal sequence (SS), KL1, KL2, and the transmembrane domain (TM). Several sheddases can cut this membrane-bound Klotho forming full-length soluble Klotho (α-cut) or KL1 and KL2 soluble Klotho (both α- and β-cut). Illustrated sequences reflect the suggested cleavage sites at which these cuts occur. Figure is created with BioRender.com.

Regarding Klotho, both ADAM10 and ADAM17 were shown to be responsible for its shedding. Chen et al. demonstrated that overexpression of either ADAM10 or ADAM17 in Klotho-transfected COS-7 cells increased release of soluble Klotho, while this shedding could be abolished by using the metalloprotease inhibitor TAPI-1 (17). Similarly, the metalloprotease inhibitor TNF484 or the ADAM10-selective inhibitor GI254023X inhibited endogenous Klotho shedding in HEK cells (18). Interestingly, besides the 130-kDa full-length ectodomain Klotho product, another smaller Klotho product of ~70-kDa could also be detected in the Klotho-expressing COS-7 cells (17). Based on the predicted primary structure it is known that the extracellular domain of Klotho consists of two tandem internal repeats, KL1 and KL2 (Figure 1), which only share 21% amino acid identity (19). As the antibodies used for Klotho detection specifically recognize the KL1 domain (20), the smaller product should correspond to the cleaved KL1 domain, which was also confirmed using mass spectrometry by other groups (17, 18). The full-length shed extracellular Klotho domain was shown to be much more abundant in the cell media than the cleaved KL1, and the cleavages that produce these forms have been termed α- and β-cut, respectively (17). For the β-cut, it could be demonstrated that membrane anchoring is essential as transfection of COS-7 cells with a truncated version of Klotho, lacking the transmembrane domain, did not generate detectable KL1 products in the medium or cell lysate (17). On the other hand, it seems that this anchoring is not necessary for the α-cut, releasing full-length Klotho in the cell media (17). Interestingly, treatment of the Klotho-expressing cells with a broad metalloprotease inhibitor Timp-3 significantly reduced the amount of KL1 not only in the medium but also in the cell lysate samples, indicating that the β-cut also takes place intracellularly (17).

To determine the exact Klotho cleavage sites of the proteinases ADAM10 and ADAM17, highly conserved regions of 34 known substrates for ADAM10 and ADAM17 (21) were analyzed and two potential recognition sites in the proximity of the Klotho transmembrane domain could be identified (22). Transfection of COS-7 cells with Klotho constructs in which these sites were mutated demonstrated that deletion of amino acids at positions 958 and 959 at the juxtamembrane site decreased soluble Klotho levels by 50–60% (22). Furthermore, deletion of the region between amino acids 954–962 almost completely abolished the presence of the 130-kDa product in the medium and the 70-kDa product in both medium and cell lysate, suggesting that not only membrane anchoring (17) but also intact α-cut sequence is required for the β-cut to occur. Overexpression of ADAM10 or ADAM17 did not result in increased shedding of the mutated Klotho, while it did in case of the intact protein, primarily when co-transfected with ADAM17 (22). In contrast to previous observations (17), however, co-transfection of the cells with ADAM10 did not increase Klotho shedding (22). Hence, it seemed that shedding by ADAM17 is prevailing over that by ADAM10 in COS-7 cells. Nonetheless, overexpression of ADAM10 in Klotho-expressing HEK293 cells did increase the amount of soluble Klotho in the medium, as shown by Bloch et al. (23). Therefore, the exact involvement of ADAM10 in Klotho shedding compared to ADAM17 remains to be further evaluated. The fact that ADAM10 is predominantly responsible for the constitutive shedding of many of its substrates, while ADAM17 is implicated in induced shedding events (24), might explain the discrepancies regarding Klotho shedding in the different studies and cell-types.

Using a similar approach, by analyzing the ADAMs' substrate compilation from Caescu et al. (21), the precise region of the β-cut could be identified as well (25). This was confirmed in COS-7 cells by transfecting the cells with Klotho mutated at the predicted β-cut site, which completely abolished KL1 fragments in the cell lysates and media (25). Moreover, the full-length Klotho product was also significantly decreased indicating that successful Klotho cleavage at either of the cuts is dependent on intact sequences at both sites, and that both α- and β-cut can occur simultaneously (25). However, it is difficult to determine whether the reduced cleavage of Klotho is caused by a mutation of the recognition sequence itself or due to potential conformational changes induced by the mutation that would render the cleavage site inaccessible for the proteinases. In either case, it also remains to be determined whether both ADAM10 and ADAM17 are responsible for both cleavages equally or if one of them is dominating in certain conditions.

Besides ADAM10 and ADAM17, Klotho was also shown to be shed by a β-secretase β-APP cleaving enzyme 1 (BACE1), as BACE1-specific siRNAs treatment of Klotho-expressing HEK293 cells resulted in a significantly decreased soluble full-length Klotho protein in the media (23). In line with this observation, overexpression of BACE1 in these cells increased the amount of shed Klotho (23). In addition, the remaining transmembrane Klotho domain is further processed by γ-secretase, since the small 5-kDa product corresponding to the Klotho stub was only visible when the cells were treated with γ-secretase inhibitors (23). Unfortunately, the exact cleavage site of BACE1 has not yet been elucidated and remains an interesting focus of future research. Nevertheless, these results are in line with previous findings that several type-I transmembrane proteins can be processed by α- and β-, as well as γ-secretases (16). Shedding by different secretases results in distinct fragments with specific properties. Such divergent effects could, for example, already be shown for amyloid precursor protein [APP; reviewed in (26)]. Shedding of APP by an α-secretase, mainly by ADAM10, generates a soluble APP fragment that has neuroprotective properties (27). In sharp contrast, shedding of APP by the β-secretase BACE1 is amyloidogenic and results in the formation of amyloid β which is a major component of amyloid plaques in Alzheimer's disease (28). Due to the similarities in Klotho and APP structure and processing, it would be highly interesting to investigate the individual roles of ADAMs and BACE1 in soluble Klotho formation and determine the presence of any functional differences between the products of the different cleavages.

## Functional Role of Soluble Klotho in Disease

### Soluble Klotho as a Biomarker for Renal Disease

As the kidney is the main source of soluble Klotho, it is not surprising that soluble levels of Klotho drastically drop in patients with CKD. Soluble Klotho levels have been observed to decrease in both blood and urine upon CKD progression (29, 30). Strikingly, this drop already occurs very early in disease development, in CKD stage 2 and often even already in CKD stage 1 (31). Associated with the reduced soluble Klotho levels, FGF23 and mineral parameters increase during CKD development (32, 33). Although the decreased soluble levels have been attributed to decreased expression of membrane Klotho (32), it may also arise from reduced shedding capacity, however this fact remains to be determined. In line with an important local role for soluble Klotho in the kidney, higher soluble Klotho levels are also independently associated with a lower risk of decline in renal function (34). Several studies have investigated whether soluble Klotho could be a potential biomarker for CKD or at least for impaired kidney function (8). However, so far, the outcomes are rather contradictory and therefore not conclusive, especially since mostly small cohorts were investigated. Another limitation for this research field is the fact that soluble Klotho is difficult to reliably measure in patient material (8), as for example, Klotho levels seem to be correlated with age (35). Furthermore, it is not possible to distinguish between full length soluble Klotho and the KL1 or KL2 fragments, although it remains to be elucidated whether these different products exert distinct functions. Therefore, further studies and larger cohorts need to be screened before drawing conclusions about the potential use of soluble Klotho as biomarker.

### Clinical Relevance of Klotho for Renal Disease in Experimental Mouse Models

Klotho was originally discovered by Kuro-o et al. in mutant mice that exhibited severe manifestations of premature aging and significantly shortened life expectancy (19). Full Klotho knockout mice (Klotho^−/−^) do not display any abnormalities until week 3–4 of age, however further development is arrested at this stage and mice generally die at the age of 8–9 weeks. Klotho^−/−^ mice also exhibit cardiac dysfunction, sterility, skin atrophy, Monckeberg type arteriosclerosis, ectopic calcifications as well as a decline in renal function (36, 37). Full Klotho knockout mice are too fragile and rarely survive surgery (38), which makes them a difficult model to work with. Notwithstanding, heterozygous Klotho-deficient mice (Klotho^+/−^) display a less striking phenotype and their life expectancy is comparable to wild type mice. At a later age, however, Klotho^+/−^ mice develop impaired kidney function with glomerulosclerosis, interstitial fibrosis and increased albuminuria (39, 40). These mice were shown to be more prone to develop a pathological response to injury, such as unilateral ureteral obstruction (UUO) or bilateral ischemia-reperfusion injury (IRI), which significantly exacerbated kidney fibrosis in the Klotho^+/−^ mice compared to wild type mice (41, 42). On the other hand, mice ubiquitously overexpressing Klotho seem to be protected against renal function deterioration in case of acute kidney injury (AKI) as well as in a glomerulonephritis model (42–44). Moreover, using adeno-associated virus (AAV)-mediated gene transfer of Klotho it could be observed that delivery of Klotho has beneficial effects in not only AKI, but also in CKD models (45, 46). Additionally, administration of recombinant soluble Klotho showed comparable effects as it reduced renal fibrosis in AKI and UUO models, suggesting these effects are primarily driven by soluble Klotho (29, 47–49). Besides local renal functions, soluble Klotho has also been shown to have systemic impact on the cardiovascular system like for example on vascular calcification as described below. A short overview of mouse models used for studying the effects of Klotho on renal and cardiovascular system is given in Table 1. These models highlight the crucial role of Klotho protein in maintaining normal functioning of the kidneys, cardiovascular system, as well as the organism as a whole. However, it remains challenging to differentiate the effects of the membrane bound/soluble full length or soluble Klotho fragments based solely on the phenotype of the Klotho mouse models. More intricate analyses might be required.

**Table 1:** Klotho mouse models for renal and cardiovascular diseases.

	**Mouse model**	**Reported outcome**	**References**
Klotho in renal disease	Aged Klotho^+/−^ mice	Impaired kidney function with glomerulosclerosis, interstitial fibrosis and increased albuminuria	(39, 40)
	UUO in Klotho^+/−^ mice	Exacerbated kidney fibrosis	(41)
	UUO in Klotho tg mice UUO in Klotho^+/−^ mice	Reduced tubulointerstitial fibrosis Enhanced tubulointerstitial fibrosis	(44)
	Recombinant Klotho treatment in UUO	Alleviation of UUO-induced EndoMT, reduced fibrosis, and improved kidney function	(48)
	Bilateral IRI in Klotho^+/−^ mice IRI in Klotho tg mice Recombinant Klotho treatment after AKI in mice or rats	Faster progression to CKD Improved preservation of kidney function after AKI Accelerated recovery and reduced renal fibrosis	(29, 42)
	Adenoviral delivery of Klotho in rats with IRI	Reduced renal damage	(46)
	ICR-derived glomerulonephritis in Klotho transgenic mice	Improved renal function and survival	(43)
Klotho in cardiovascular complications	Klotho^+/−^ mice or tg mice with uni-Nx with IRI in contralateral kidney	Reduced or improved renal function and vascular calcification, respectively	(37)
	Klotho^−/−^ mice with diabetic nephropathy AAV-mediated delivery of soluble Klotho	Hyperphosphatemia and enhanced vascular calcification Rescued phosphate levels and prevention of calcification	(45)
	Hind limb ischemia in Klotho^−/−^ and Klotho^+/−^ mice	Impaired angiogenesis and vasculogenesis	(50)
	Klotho^−/−^ and Klotho^+/−^ mice	Impaired vasodilation/vasorelaxation, rescued by parabiosis with wt mice	(51)
	Klotho^+/−^ mice	Cardiac dysfunction, hypertrophy and fibrosis	(52)
	Klotho administration in mice with uni-Nx with IRI in contralateral kidney	Attenuated CKD-associated cardiac remodeling	(49)

*AKI, acute kidney injury; CKD, chronic kidney disease; EndoMT, endothelial-to-mesenchymal transition; IRI, ischemia-reperfusion injury; Nx, nephrectomy; tg, transgenic; UUO, unilateral ureteral obstruction; wt, wild type*.

### Klotho in Cardiovascular Complications of Renal Disease

Vascular calcification appears early in the course of CKD and becomes more prevalent as kidney function decreases and thereby causes a high risk of cardiovascular mortality in patients with CKD (4, 53). Obviously, considering its major role in regulating mineral (Ca/phosphate) homeostasis, Klotho deficiency causes high circulating phosphate levels and thereby strongly enhances vascular calcification in mice with CKD (37). Recently, it could be shown that delivery of AAV expressing soluble Klotho into Klotho deficient mice reduces phosphate levels and, in line with this, vascular calcification (45). Moreover, Klotho deficiency in CKD enhances renal tubule and vascular cell senescence which impairs angiogenesis and vasculogenesis (50). Together, these results clearly demonstrate that Klotho plays a protective role in vascular calcification and CKD, although cell-specific effects remain rather elusive. These protective effects of Klotho are probably mostly indirect in nature as they are related to its ability to regulate the effects of several growth factors, such as FGF23, and ion-channels, as discussed before. However, soluble Klotho also suppresses the activity of the WNT/β-catenin pathway in stem and progenitor cells in a direct manner, which has been shown to be important not only for vascular calcification, but also aging (54). Interestingly, WNT/β-catenin, in turn, inhibits renal Klotho expression. Via this loop Klotho and WNT signaling interact and play an important role in CKD and associated complications (55).

Furthermore, elevated levels of soluble Klotho in plasma are independently associated with a lower risk of cardiovascular disease (56). This can at least partly be explained by the observed vasculoprotective effects of soluble Klotho on the endothelium, as production of nitric oxide and vasodilation are impaired in heterozygous Klotho deficient rodent (51). Additionally, soluble Klotho has been identified as an anti-inflammatory modulator, since a bidirectional negative relationship between Klotho and NF-κB could be identified in which Klotho impairs translocation and hence activation of NF-κB in cultured endothelial cells (57). Thereby Klotho also suppresses expression of the adhesion molecules intercellular adhesion molecule 1 (ICAM-1) and vascular cell adhesion molecule 1 (VCAM-1) in endothelial cells (57). Klotho also reduces the expression of lectin-like oxidized low-density lipoprotein receptor-1 (LOX-1), a major receptor for oxidized LDL, in cultured endothelial cells (58). However, the direct impact on vascular cells and the exact mechanism of action remains poorly understood, especially as receptors that mediate the effects remain largely unknown and the effects of soluble Klotho seem to be at least partially FGFR23 independent (59). Not surprisingly, as all of the described mechanisms play a crucial role in atherosclerosis development, it could be shown that lower levels of serum soluble Klotho were associated with increased carotid artery intima-media thickness and could thereby be considered an early predictor of atherosclerosis (56, 60). Although the ectopic expression of Klotho is still under debate, recent studies demonstrate its expression in cardiomyocytes and highlight the impact of Klotho on cardiac diseases, like myocardial infarction and left ventricular hypertrophy [as reviewed in (61, 62)]. In line with this, subjects at high risk for atherosclerotic/cardiovascular events have a reduced expression of Klotho in cardiomyocytes (63), associated with increased oxidative stress, inflammation and fibrosis, although the direct impact of Klotho on cardiomyocytes has not been examined in this study.

## Discussion and Future Directions

Over the course of more than two decades after the serendipitous identification of the Klotho protein (19), research has focused on elucidating the exact function of this protein in health and disease. It has already been described that Klotho plays a role in a multitude of processes and this list will only grow further over time. At the moment, the role of Klotho in aging, kidney disease, more particularly CKD, and the vasculature is quite well described. Yet, the direct vs. indirect functions of soluble Klotho on vascular cells, receptor(s) involved, and the exact underlying mechanisms of action remain largely unknown or contradictory and should therefore be a focus of future research.

Recently, it was demonstrated that all the soluble Klotho arises from shedding of membrane-bound Klotho as the alternatively spliced variant is subject to nonsense-mediated mRNA decay and degraded (15). While ADAM10, ADAM17 as well as BACE have been implicated in klotho shedding, other proteases may be involved as well. Shedding of Klotho can result in different fragments, being either a full-length fragment or smaller sub-fragments called KL1 and KL2. However, until now most studies did not clearly distinguish between these different fragments or mainly used full-length soluble Klotho. Therefore, further studies are needed to elucidate which fragments are produced by the different shedding enzymes and determine the specific functional implication of the cleavage process resulting in KL1 and KL2 fragments.

In order to fully comprehend the function of soluble Klotho and to enable potential therapeutic targeting it is highly important that future research focuses on the elucidation of the exact underlying mechanisms. Only recently the crystal structure of Klotho has been described elucidating the exact structure of Klotho protein (64). This might give an important impulse to the research field. Recently, a potential mechanism of action of soluble Klotho has been suggested, as it could be identified that soluble Klotho binds to membrane lipid rafts which alters the lipid organization in the cell membrane (65, 66). However, further functional studies are needed to elucidate the importance of this interaction.

**Figure 2:**
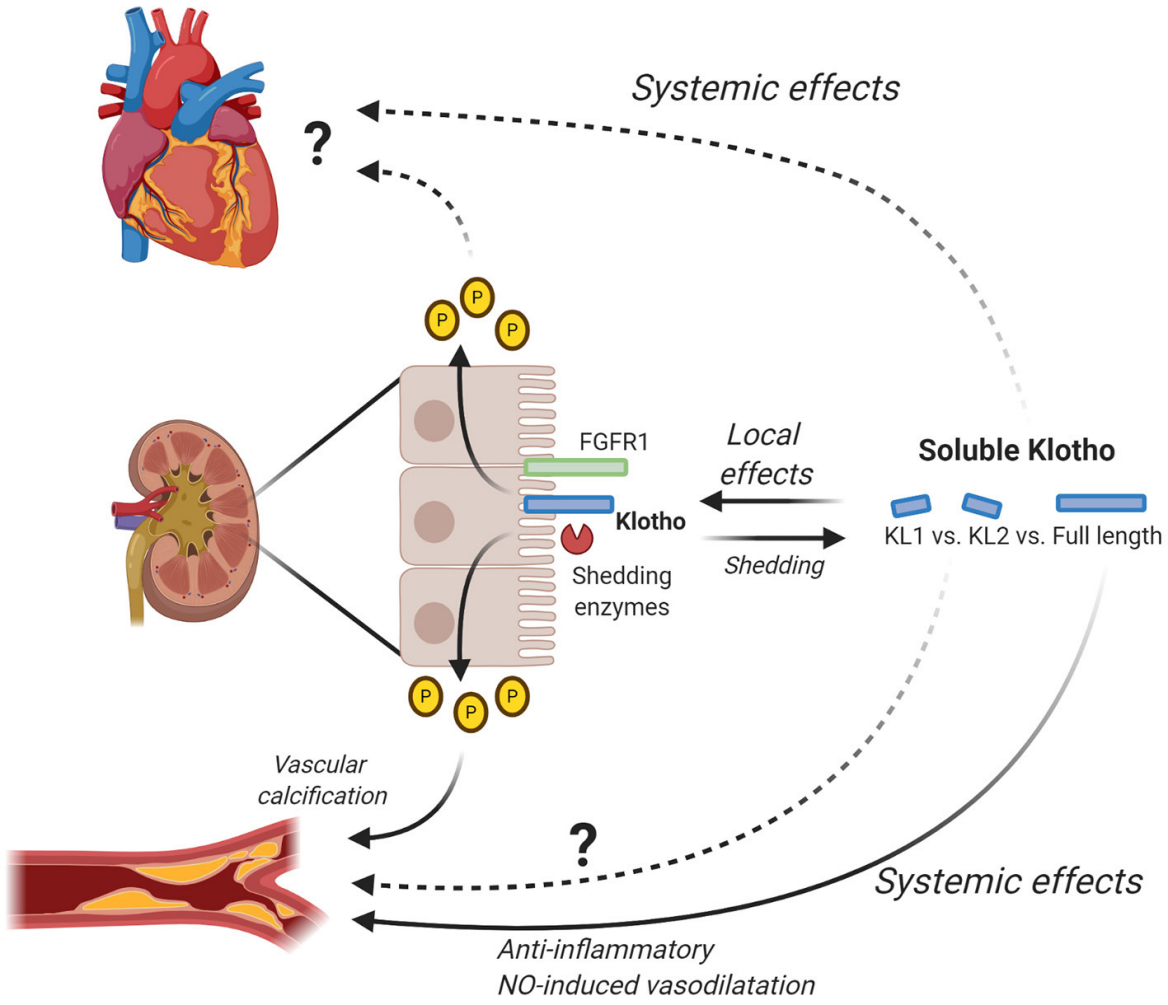
Local and systemic effects of soluble Klotho. Klotho is mainly expressed in the kidney where it interacts with FGF23 to regulate phosphate levels. By changing phosphate levels, Klotho for example has an effect on vascular calcification and possibly also on the heart. Additionally, Klotho is shed by shedding enzymes (e.g., ADAM10/ADAM17) generating soluble Klotho, either full-length or subfragment KL1 and KL2. Soluble Klotho has local effects in the kidney where it contributes to kidney function and in relation to this also CKD. Full-length soluble Klotho has been shown to have systemic effects on the vascular system by inducing anti-inflammatory effects and NO-induced vasodilatation. The exact effects of KL1 and KL2 on the vascular system remain to be determined. Furthermore, further research is needed to elucidate the exact systemic effects of full-length soluble Klotho as well as KL1 and KL2 on the heart. Figure is created with BioRender.com.

In conclusion, soluble Klotho plays an important role in health and disease (Figure 2) and is thereby a promising therapeutic target. However, further research is first needed to improve our understanding of the exact effects and especially the regulation of Klotho shedding.

## Author Contributions

VS-K wrote the manuscript. MD made the critical revisions to the manuscript. EV wrote the manuscript. All authors contributed to the article and approved the submitted version.

## Conflict of Interest

The authors declare that the research was conducted in the absence of any commercial or financial relationships that could be construed as a potential conflict of interest.

## References

[1] Wang Y Kuro-o M Sun Z. Current understanding of klotho. Ageing Res Rev. (2009) 8:43–51. 10.1016/j.arr.2008.10.002 19022406 PMC2637560

[2] Dalton GD Xie J An SW Huang CL. New insights into the mechanism of action of soluble klotho. Front Endocrinol (Lausanne). (2017) 8:323. 10.3389/fendo.2017.00323 29250031 PMC5715364

[3] Kuro-o M. The Klotho proteins in health and disease. Nat Rev Nephrol. (2019) 15:27–44. 10.1038/s41581-018-0078-3 30455427

[4] Buchanan S Combet E Stenvinkel P Shiels PG. Klotho, aging, and the failing kidney. Front Endocrinol (Lausanne). (2020) 11:560. 10.3389/fendo.2020.00560 32982966 PMC7481361

[5] Zhu L Stein LR Kim D Ho K Yu GQ Zhan L. Klotho controls the brain–immune system interface in the choroid plexus. Proc Natl Acad Sci USA. (2018) 115:E11388–96. 10.1073/pnas.1808609115 30413620 PMC6275534

[6] Hu MC Shiizaki K Kuro-O M Moe OW. Fibroblast growth factor 23 and klotho: physiology and pathophysiology of an endocrine network of mineral metabolism. Annu Rev Physiol. (2013) 75:503–33. 10.1146/annurev-physiol-030212-183727 23398153 PMC3770142

[7] Bär L Stournaras C Lang F Föller M. Regulation of fibroblast growth factor 23 (FGF23) in health and disease. FEBS Lett. (2019) 593:1879–900. 10.1002/1873-3468.13494 31199502

[8] Zou D Wu W He Y Ma S Gao J. The role of klotho in chronic kidney disease. BMC Nephrol. (2018) 19:285. 10.1186/s12882-018-1094-z 30348110 PMC6198535

[9] Razzaque MS. The FGF23-Klotho axis: endocrine regulation of phosphate homeostasis. Nat Rev Endocrinol. (2009) 5:611–9. 10.1038/nrendo.2009.196 19844248 PMC3107967

[10] Shimada T Kakitani M Yamazaki Y Hasegawa H Takeuchi Y Fujita T. Targeted ablation of Fgf23 demonstrates an essential physiological role of FGF23 in phosphate and vitamin D metabolism. J Clin Invest. (2004) 113:561–8. 10.1172/jci19081 14966565 PMC338262

[11] Stubbs JR Liu S Tang W Zhou J Wang Y Yao X. Role of hyperphosphatemia and 1,25-dihydroxyvitamin D in vascular calcification and mortality in fibroblastic growth factor 23 null mice. J Am Soc Nephrol. (2007) 18:2116–24. 10.1681/ASN.2006121385 17554146

[12] Hu MC Shi M Zhang J Addo T Cho HJ Barker SL. Renal production, uptake, and handling of circulating αklotho. J Am Soc Nephrol. (2016) 27:79–90. 10.1681/ASN.2014101030 25977312 PMC4696570

[13] Imura A Iwano A Tohyama O Tsuji Y Nozaki K Hashimoto N. Secreted Klotho protein in sera and CSF: implication for post-translational cleavage in release of Klotho protein from cell membrane. FEBS Lett. (2004) 565:143–7. 10.1016/j.febslet.2004.03.090 15135068

[14] Matsumura Y Aizawa H Shiraki-Iida T Nagai R Kuro-O M Nabeshima YI. Identification of the human klotho gene and its two transcripts encoding membrane and secreted klotho protein. Biochem Biophys Res Commun. (1998) 242:626–30. 10.1006/bbrc.1997.8019 9464267

[15] Mencke R Harms G Moser J van Meurs M Diepstra A Leuvenink HG. Human alternative Klotho mRNA is a nonsense-mediated mRNA decay target inefficiently spliced in renal disease. JCI Insight. (2017) 2:e94375. 10.1172/jci.insight.94375 29046474 PMC5846909

[16] Lichtenthaler SF Lemberg MK Fluhrer R. Proteolytic ectodomain shedding of membrane proteins in mammals—hardware, concepts, and recent developments. EMBO J. (2018) 37:99456. 10.15252/embj.201899456 29976761 PMC6068445

[17] Chen C Di Podvin S Gillespie E Leeman SE Abraham CR. Insulin stimulates the cleavage and release of the extracellular domain of Klotho by ADAM10 and ADAM17. Proc Natl Acad Sci USA. (2007) 104:19796–801. 10.1073/pnas.0709805104 18056631 PMC2148378

[18] van Loon EPMM Pulskens WP Van Der Hagen EAEE Lavrijsen M Vervloet MG Van Goor H. Shedding of klotho by ADAMs in the kidney. Am J Physiol Ren Physiol. (2015) 309:F359–68. 10.1152/ajprenal.00240.2014 26155844

[19] Kuro-o M Matsumura Y Aizawa H Kawaguchi H Suga T Utsugi T. Mutation of the mouse klotho gene leads to a syndrome resembling ageing. Nature. (1997) 390:45–51. 10.1038/36285 9363890

[20] Kato Y Arakawa E Kinoshita S Shirai A Furuya A Yamano K. Establishment of the anti-klotho monoclonal antibodies and detection of klotho protein in kidneys. Biochem Biophys Res Commun. (2000) 267:597–602. 10.1006/bbrc.1999.2009 10631108

[21] Caescu CI Jeschke GR Turk BE. Active-site determinants of substrate recognition by the metalloproteinases TACE and ADAM10. Biochem J. (2009) 424:79–88. 10.1042/BJ20090549 19715556 PMC2774824

[22] Chen C Di Tung TY Liang J Zeldich E Tucker Zhou TB Turk BE. Identification of cleavage sites leading to the shed form of the anti-aging protein klotho. Biochemistry. (2014) 53:5579–87. 10.1021/bi500409n 25110992 PMC4151695

[23] Bloch L Sineshchekova O Reichenbach D Reiss K Saftig P Kuro-o M. Klotho is a substrate for α-, β- and γ-secretase. FEBS Lett. (2009) 583:3221–4. 10.1016/j.febslet.2009.09.009 19737556 PMC2757472

[24] van der Vorst E Weber C Donners M. A disintegrin and metalloproteases (ADAMs) in cardiovascular, metabolic and inflammatory diseases: aspects for theranostic approaches. Thromb Haemost. (2018) 118:1167–75. 10.1055/s-0038-1660479 29874690

[25] Chen C Di Li Y Chen AK Rudy MA Nasse JS Zeldich E. Identification of the cleavage sites leading to the shed forms of human and mouse antiaging and cognition-enhancing protein Klotho. PLoS ONE. (2020) 15:e0226382. 10.1371/journal.pone.0226382 31929539 PMC6957300

[26] Zheng H Koo EH. The amyloid precursor protein: beyond amyloid. Mol Neurodegener. (2006) 1:5. 10.1186/1750-1326-1-5 16930452 PMC1538601

[27] Chasseigneaux S Allinquant B. Functions of Aβ, sAPPα and sAPPβ: similarities and differences. J Neurochem. (2012) 120:99–108. 10.1111/j.1471-4159.2011.07584.x 22150401

[28] Cole SL Vassar R. The role of amyloid precursor protein processing by BACE1, the β-secretase, in Alzheimer disease pathophysiology. J Biol Chem. (2008) 283:29621–5. 10.1074/jbc.R800015200 18650431 PMC2662048

[29] Hu MC Shi M Zhang J Quiones H Kuro-O M Moe OW. Klotho deficiency is an early biomarker of renal ischemia-reperfusion injury and its replacement is protective. Kidney Int. (2010) 78:1240–51. 10.1038/ki.2010.328 20861825 PMC3237296

[30] Izquierdo MC Perez-Gomez M V. Sanchez-Niño MD Sanz AB Ruiz-Andres O Poveda J. Klotho, phosphate and inflammation/ageing in chronic kidney disease. Nephrol Dial Transplant. (2012) 27:iv6–10. 10.1093/ndt/gfs426 23258814

[31] Pavik I Jaeger P Ebner L Wagner CA Petzold K Spichtig D. Secreted Klotho and FGF23 in chronic kidney disease stage 1 to 5: a sequence suggested from a cross-sectional study. Nephrol Dial Transplant. (2013) 28:352–9. 10.1093/ndt/gfs460 23129826

[32] Barker SL Pastor J Carranza D Quiones H Griffith C Goetz R. The demonstration of αKlotho deficiency in human chronic kidney disease with a novel synthetic antibody. Nephrol Dial Transplant. (2015) 30:223–33. 10.1093/ndt/gfu291 25324355 PMC4309192

[33] Dhayat NA Ackermann D Pruijm M Ponte B Ehret G Guessous I. Fibroblast growth factor 23 and markers of mineral metabolism in individuals with preserved renal function. Kidney Int. (2016) 90:648–57. 10.1016/j.kint.2016.04.024 27370409

[34] Drew DA Katz R Kritchevsky S Ix J Shlipak M Gutiérrez OM. Association between soluble klotho and change in kidney function: the health aging and body composition study. J Am Soc Nephrol. (2017) 28:1859–66. 10.1681/ASN.2016080828 28104822 PMC5461794

[35] Yamazaki Y Imura A Urakawa I Shimada T Murakami J Aono Y. Establishment of sandwich ELISA for soluble alpha-Klotho measurement: age-dependent change of soluble alpha-Klotho levels in healthy subjects. Biochem Biophys Res Commun. (2010) 398:513–8. 10.1016/j.bbrc.2010.06.110 20599764 PMC4130489

[36] Nabeshima YI Klotho deficient mouse: an *in vivo* model for human aging. Drug Discov Today Dis Model. (2004) 1:223–7. 10.1016/j.ddmod.2004.11.023

[37] Hu MC Shi M Zhang J Quiñones H Griffith C Kuro-o M. Klotho deficiency causes vascular calcification in chronic kidney disease. J Am Soc Nephrol. (2011) 22:124–36. 10.1681/ASN.2009121311 21115613 PMC3014041

[38] Mencke R Olauson H Hillebrands JL. Effects of Klotho on fibrosis and cancer: a renal focus on mechanisms and therapeutic strategies. Adv Drug Deliv Rev. (2017) 121:85–100. 10.1016/j.addr.2017.07.009 28709936

[39] Zhou X Chen K Lei H Sun Z. Klotho gene deficiency causes salt-sensitive hypertension via monocyte chemotactic protein-1/CC chemokine receptor 2-mediated inflammation. J Am Soc Nephrol. (2015) 26:121–32. 10.1681/ASN.2013101033 24904083 PMC4279730

[40] Zhou X Chen K Wang Y Schuman M Lei H Sun Z. Antiaging gene klotho regulates adrenal CYP11B2 expression and aldosterone synthesis. J Am Soc Nephrol. (2016) 27:1765–76. 10.1681/ASN.2015010093 26471128 PMC4884100

[41] Sugiura H Yoshida T Shiohira S Kohei J Mitobe M Kurosu H. Reduced klotho expression level in kidney aggravates renal interstitial fibrosis. Am J Physiol Ren Physiol. (2012) 302:1252–64. 10.1152/ajprenal.00294.2011 22338084

[42] Shi M Flores B Gillings N Bian A Cho HJ Yan S. Aklotho mitigates progression of Aki to Ckd through activation of autophagy. J Am Soc Nephrol. (2016) 27:2331–45. 10.1681/ASN.2015060613 26701976 PMC4978045

[43] Haruna Y Kashihara N Satoh M Tomita N Namikoshi T Sasaki T. Amelioration of progressive renal injury by genetic manipulation of Klotho gene. Proc Natl Acad Sci USA. (2007) 104:2331–6. 10.1073/pnas.0611079104 17287345 PMC1892974

[44] Satoh M Nagasu H Morita Y Yamaguchi TP Kanwar YS Kashihara N. Klotho protects against mouse renal fibrosis by inhibiting Wnt signaling. Am J Physiol Ren Physiol. (2012) 303:1641–51. 10.1152/ajprenal.00460.2012 23034937 PMC3532475

[45] Hum JM O'Bryan LM Tatiparthi AK Cass TA Clinkenbeard EL Cramer MS. Chronic hyperphosphatemia and vascular calcification are reduced by stable delivery of soluble klotho. J Am Soc Nephrol. (2017) 28:1162–74. 10.1681/ASN.2015111266 27837149 PMC5373441

[46] Sugiura H Yoshida T Tsuchiya K Mitobe M Nishimura S Shirota S. Klotho reduces apoptosis in experimental ischaemic acute renal failure. Nephrol Dial Transplant. (2005) 20:2636–45. 10.1093/ndt/gfi165 16204278

[47] Doi S Zou Y Togao O Pastor J V. John GB Wang L. Klotho inhibits transforming growth factor-β1 (TGF-β1) signaling and suppresses renal fibrosis and cancer metastasis in mice. J Biol Chem. (2011) 286:8655–65. 10.1074/jbc.M110.174037 21209102 PMC3048747

[48] Li S Yu L He A Liu Q. Klotho inhibits unilateral ureteral obstruction-induced endothelial-to-mesenchymal transition via TGF-β1/Smad2/Snail1 signaling in mice. Front Pharmacol. (2019) 10:348. 10.3389/fphar.2019.00348 31024315 PMC6460315

[49] Hu MC Shi M Gillings N Flores B Takahashi M Kuro-o M. Recombinant α-Klotho may be prophylactic and therapeutic for acute to chronic kidney disease progression and uremic cardiomyopathy. Kidney Int. (2017) 91:1104–14. 10.1016/j.kint.2016.10.034 28131398 PMC5592833

[50] Shimada T Takeshita Y Murohara T Sasaki KI Egami K Shintani S. Angiogenesis and vasculogenesis are impaired in the precocious-aging klotho mouse. Circulation. (2004) 110:1148–55. 10.1161/01.CIR.0000139854.74847.99 15302783

[51] Saito Y Yamagishi T Nakamura T Ohyama Y Aizawa H Suga T. Klotho protein protects against endothelial dysfunction. Biochem Biophys Res Commun. (1998) 248:324–9. 10.1006/bbrc.1998.8943 9675134

[52] Hu MC Shi M Cho HJ Adams-Huet B Paek J Hill K. Klotho and phosphate are modulators of pathologic uremic cardiac remodeling. J Am Soc Nephrol. (2015) 26:1290–302. 10.1681/ASN.2014050465 25326585 PMC4446876

[53] Vervloet MG Adema AY Larsson TE Massy ZA. The role of Klotho on vascular calcification and endothelial function in chronic kidney disease. Semin Nephrol. (2014) 34:578–85. 10.1016/j.semnephrol.2014.09.003 25498377

[54] Liu H Fergusson MM Castilho RM Liu J Cao L Chen J. Augmented Wnt signaling in a mammalian model of accelerated aging. Science. (2007) 317:803–6. 10.1126/science.1143578 17690294

[55] Muñoz-Castañeda JR Rodelo-Haad C Pendon-Ruiz de Mier MV Martin-Malo A Santamaria R Rodriguez M. Klotho/FGF23 and Wnt signaling as important players in the comorbidities associated with chronic kidney disease. Toxins (Basel). (2020) 12:185. 10.3390/toxins12030185 32188018 PMC7150840

[56] Semba RD Cappola AR Sun K Bandinelli S Dalal M Crasto C. Plasma klotho and cardiovascular disease in adults. J Am Geriatr Soc. (2011) 59:1596–601. 10.1111/j.1532-5415.2011.03558.x 21883107 PMC3486641

[57] Maekawa Y Ishikawa K Yasuda O Oguro R Hanasaki H Kida I. Klotho suppresses TNF-α-induced expression of adhesion molecules in the endothelium and attenuates NF-κB activation. Endocrine. (2009) 35:341–6. 10.1007/s12020-009-9181-3 19367378

[58] Yao Y Wang Y Zhang Y Liu C. Klotho ameliorates oxidized low density lipoprotein (ox-LDL)-induced oxidative stress via regulating LOX-1 and PI3K/Akt/eNOS pathways. Lipids Health Dis. (2017) 16:77. 10.1186/s12944-017-0447-0 28407763 PMC5390438

[59] Quarles LD. Fibroblast growth factor 23 and α-Klotho co-dependent and independent functions. Curr Opin Nephrol Hypertens. (2019) 28:16–25. 10.1097/MNH.0000000000000467 30451736 PMC6258326

[60] Keles N Caliskan M Dogan B Keles NN Kalcik M Aksu F. Low serum level of Klotho is an early predictor of atherosclerosis. Tohoku J Exp Med. (2015) 237:17–23. 10.1620/tjem.237.17 26289053

[61] Olejnik A Franczak A Krzywonos-Zawadzka A Kałuzna-Oleksy M Bil-Lula I. The biological role of klotho protein in the development of cardiovascular diseases. Biomed Res Int. (2018) 2018:17. 10.1155/2018/5171945 30671457 PMC6323445

[62] Navarro-García JA Fernández-Velasco M Delgado C Delgado JF Kuro-o M Ruilope LM. PTH, vitamin D, and the FGF-23–klotho axis and heart: going beyond the confines of nephrology. Eur J Clin Invest. (2018) 48:e12902. 10.1111/eci.12902 29394451

[63] Corsetti G Pasini E Scarabelli TM Romano C Agrawal PR Chen-Scarabelli C. Decreased expression of Klotho in cardiac atria biopsy samples from patients at higher risk of atherosclerotic cardiovascular disease. J Geriatr Cardiol. (2016) 13:701–11. 10.11909/j.issn.1671-5411.2016.08.009 27781061 PMC5067432

[64] Chen G Liu Y Goetz R Fu L Jayaraman S Hu MC. α-Klotho is a non-enzymatic molecular scaffold for FGF23 hormone signalling. Nature. (2018) 553:461–6. 10.1038/nature25451 29342138 PMC6007875

[65] Dalton G An SW Al-Juboori SI Nischan N Yoon J Dobrinskikh E. Soluble klotho binds monosialoganglioside to regulate membrane microdomains and growth factor signaling. Proc Natl Acad Sci USA. (2017) 114:752–7. 10.1073/pnas.1620301114 28069944 PMC5278494

[66] Wright JD An SW Xie J Yoon J Nischan N Kohler JJ. Modeled structural basis for the recognition of a2-3-sialyllactose by soluble Klotho. FASEB J. (2017) 31:3574–86. 10.1096/fj.201700043R 28442546 PMC5503716

